# Aneurism of the Abdominal Aorta

**Published:** 1855-09

**Authors:** 


Aneurism of the Abdominal Aorta.—1. That aneurism of the abdominal aorta, especially when it arrives high up in the course of the vessel, may be attended with severe and peculiar neuralgic pains.

2. That in certain cases these pains are so characteristic as to enable us to diagnosticate the disease with probable accuracy.

3. That they may exist for a long period without any symptom of constitutional ailment.

4. That they cannot be attributed solely to erosion of the vertebræ.

5. That aneurism in the abdomen is less often connected with extensive arterial lesion than when it occurs in the thorax.

6. That the want of proportion between the intensity of the sufferings and the amount of constitutional disturbance is often an important source of diagnosis.

7. That although the occurrence of constitutional suffering is generally seen when the aneurism becomes diffused, yet that in some cases it seems to attend the earlier stages of the disease.

8. That fever is absent in the earlier stages.

9. That fever is rarely met with in any stage of abdominal aneurism. It is absent in the first stages before the aneurism becomes diffused; and when appearing after that change, it is of a low, irritative type.

10. That the pulsations in the disease are generally single, and that they are more certainly attended with bellows murmur than those which accompany thoracic aneurism.

11. That displacement of important organs may long precede the fatal rupture or the diffusion of the aneurism.

12. That the pulsations of the tumor are in most cases single, but that in a case where the aneurism was seated high up a double pulsation has been observed.

13. That abdominal aneurism may cause displacement of the heart to the right side.

14. That the murmur has been known to disappear in the erect position.

15. That true aneurism of the abdominal aorta is rarely met with.

16. That death may occur from the rupture of the original sac into the peritoneal or pleural cavity; by the aneurism becoming diffused (secondary false aneurism); or by simple exhaustion of the patient, without rupture of the sac.

17. That the patient often obtains relief by lying on his face: in one case all signs of the tumor disappeared on turning to the left side.

18. That sudden and fatal hemorrhage may proceed from the opening of the diffused aneurism into a serous sac.

19. That the diffusion of the aneurism is often indicated, not only by a remarkable change in the constitutional state, but by alterations of the physical phenomena of the sac and of the heart.

20. That great tenderness of the belly may be thus produced, and yet no peritoneal inflammation exist.

21. That local suffering may attend the occurrence of the effusion.

22. That the new deposites of blood may present a feeble diastolic pulsation and a soft and diffused bellows murmur; in some cases, however, they appear as non-pulsating masses, or receive a communicated stroke from the subjacent or contiguous artery.

23. That, as a general rule, we find the force of the heart, and that of the throbbings in the original sac, diminish when the aneurism becomes diffused. In one case, however, excitement, both of the heart and aneurism, preceded the accident.

24. That in cases where much blood has been effused, the sounds of the heart may become single, the original tumor less distinct, and the bellows murmur diminished.

25. That in these instances it appears probable that it is the systolic sound of the heart that is wanting.

26. That in a case where a copious effusion had occurred between the folds of the mesentery, two murmurs, differing in seat, tone, and quality were observed.

27. That all the solid viscera of the abdomen are liable to be displaced in the course of the disease.

28. That the violent neuralgic pains are not necessarily connected with absorption of the vertebræ; and that this lesion may be found without even paraoxysmal or constant pain.

29. That two forms of pains are often met with—one violent, neuralgic, and paroxysmal; the other more dull, deep-seated, and boring.

30. That the tumor formed by a non-diffused aneurism is immovable.

31. That the first development of a murmur low down in the abdomen should incline us against the diagnosis of aneurism.

32. That the abdominal tumors which most simulate aneurism are those whose consistence is semi-fluid.

33. That when these tumors are movable, the pulsation, and even the murmur, can be made to appear and disappear.

34. That while the progress of an aneurismal tumor is generally from above downwards, that of the solid tumors is more often from below upward.

35. That the first appearance of pulsation, at some point low down in the belly, indicates that it is communicated to rather than inherent in the tumor.

36. That there are three important conditions occasionally attendant upon solid tumors of the belly which we have never observed in aneurism. These are—

*a.* Collateral venous circulation, as shown by the enlargement of the epigastric veins.

*b.* The existence of ascitas.

*c.* The occurrence of friction sound and vibration ovor the tumor.

37. That in cases of permanent patency of aortic valves, a temporary increase of the pulsations of the abdominal aorta, probably induced by sympathetic irritation, has led to the erroneous diagnosis of abdominal aneurism.

38. That a tumor with fluid contents, such as an hepatic abscess, may have completely diastolic pulsation. Here the diagnosis will depend on the preceding and accompanying circumstances of the case.—*Ibid.*

